# Pharmacokinetic and exposure–response analyses of pertuzumab in combination with trastuzumab and docetaxel during neoadjuvant treatment of HER2+ early breast cancer

**DOI:** 10.1007/s00280-016-3218-0

**Published:** 2017-01-10

**Authors:** Angelica L. Quartino, Hanbin Li, Jin Y. Jin, D. Russell Wada, Mark C. Benyunes, Virginia McNally, Lucia Viganò, Ihsan Nijem, Bert L. Lum, Amit Garg

**Affiliations:** 1 Genentech, Inc., South San Francisco, CA USA; 2 Quantitative Solutions/Certara, Menlo Park, CA USA; 3 Roche Products Limited, Welwyn, UK; 4 San Raffaele Hospital – Research Institute, Milan, Italy

**Keywords:** Pertuzumab, Exposure–response, Pharmacokinetics, Early breast cancer, HER2, Neoadjuvant

## Abstract

**Purpose:**

The NeoSphere trial evaluated pertuzumab in the neoadjuvant setting [early breast cancer (EBC)] with pathological complete response (pCR) as the primary efficacy end point. This analysis of pertuzumab aimed to (1) compare its pharmacokinetics (PK) in patients with EBC versus advanced cancers, (2) to further evaluate PK drug–drug interactions (DDIs) when given in combination with trastuzumab, and (3) to assess the relationship between exposure and efficacy to assess the clinical dosing regimen in the EBC patients.

**Methods:**

Pertuzumab serum concentration data from 180 patients in NeoSphere were compared to historical observations and potential DDI was assessed, by applying simulation techniques using a population PK model. The impact of pertuzumab exposure on pCR rate was evaluated using a logit response model (*n* = 88).

**Results:**

The observed PK matched the population PK model simulations, confirming that the PK in neoadjuvant EBC appear to be in agreement with the historical observations. No evidence of a DDI effect of trastuzumab or docetaxel on pertuzumab was observed supporting the doses when given in combination. In NeoSphere >90% of EBC patients achieved the non-clinical target serum concentration. There was no association between the pertuzumab serum concentration and pCR within the range observed in this study (20–100 μg/mL) supporting no dose adjustments needed for patients with lower exposure.

**Conclusions:**

This analysis further supports the lack of DDI between the two therapeutic proteins and the appropriateness of the approved fixed non-body-weight-adjusted pertuzumab dose in the treatment of neoadjuvant EBC with pertuzumab in combination with trastuzumab and docetaxel.

**Electronic supplementary material:**

The online version of this article (doi:10.1007/s00280-016-3218-0) contains supplementary material, which is available to authorized users.

## Introduction

Pertuzumab (PERJETA^®^, F. Hoffmann-La Roche, Basel, Switzerland) is a recombinant, humanized, immunoglobulin (Ig)G1κ monoclonal antibody, which targets human epidermal growth factor receptor 2 (HER2). Pertuzumab is the first in a new class of targeted cancer treatments called HER2 dimerization inhibitors. Non-clinical data indicate that pertuzumab and trastuzumab (Herceptin^®^, F. Hoffmann-La Roche, Basel, Switzerland) bind to distinct epitopes on the HER2 without competing with each other and have distinct mechanisms for disrupting HER2 signaling [1, 2]. These mechanisms are complementary and result in augmented anti-proliferative activity in vitro and in vivo when pertuzumab and trastuzumab are given in combination [3–5]. By binding to the subdomain II of the extracellular domain of HER2, pertuzumab prevents heterodimerization of HER2 with other members of the HER family (HER1, HER3, and HER4). As a result, ligand-activated downstream signaling is blocked by pertuzumab. Pertuzumab is also capable of activating antibody-dependent cell-mediated cytotoxicity (ADCC) similar to trastuzumab [6]. Pertuzumab in combination with trastuzumab and docetaxel was shown to significantly improve progression-free survival (PFS) and overall survival (OS) in patients with first-line metastatic HER2-positive breast cancer, which led to its approval [7] in the USA in 2012 and in the European Union in 2013 with intravenous dosing at a fixed (non-weight-based dose) loading dose of 840 mg, followed by 420 mg on a every three-week (q3w) schedule [7–11]. In the pivotal trial, CLEOPATRA, no DDI between pertuzumab and trastuzumab and between pertuzumab and docetaxel was detected in a limited number of patients evaluated [12].

NeoSphere, a Phase II, multicenter study spread across 16 countries for HER2-positive breast cancer patients, was conducted to assess the activity of pertuzumab (PERJETA^®^) by comparing the therapeutic effects of the conventional combination of trastuzumab (Herceptin^®^) plus docetaxel with the combination of pertuzumab with either docetaxel or trastuzumab, or both, in a neoadjuvant setting. This clinical trial was a four-arm study evaluating the efficacy and safety of neoadjuvant treatment regimens in female patients with locally advanced, inflammatory or early-stage HER2-positive breast cancer. Before surgery, patients were randomized to receive four cycles of one of the following four treatment arms: (A) trastuzumab + docetaxel, (B) trastuzumab + docetaxel + pertuzumab, (C) trastuzumab + pertuzumab, and (D) pertuzumab + docetaxel. Post-surgery patients in arm A, B, and D received three cycles of 5-fluorouracil, epirubicin, and cyclophosphamide (FEC) and trastuzumab to complete 1-year treatment (17 cycles in total). Patients in arm C received four cycles of docetaxel followed by three cycles of FEC and trastuzumab to complete 1-year treatment (21 cycles in total). The primary end point was pathological complete response (pCR) evaluated after Cycle 4. Pertuzumab increased the pCR response rate in patients when used in combination with trastuzumab and docetaxel (Table 1) [6]. Overall, in the NeoSphere study, a significantly higher proportion of women given neoadjuvant pertuzumab and trastuzumab plus docetaxel achieved pCR in the breast than did those given trastuzumab and docetaxel alone, leading to its approval in the USA and in the European Union in 2013 and 2015, respectively. Although pertuzumab plus docetaxel was efficacious, the combination of chemotherapy with both antibodies was more active than chemotherapy with either antibody alone [9]. A 5-year analysis showed that patients achieving a total pCR with all groups combined had a longer PFS compared with patients that did not achieve total pCR, thus suggesting that pCR could be an early indicator of long-term outcome in early-stage HER2-positive breast cancer [13].

**Table 1:** Covariates and pCR response by treatment group in NeoSphere

Treatment group	A	B	C	D	Total
Trastuzumab	**+**	**+**	**+**	**−**	
Docetaxel	**+**	**+**	**−**	**+**	
Pertuzumab	**−**	**+**	**+**	**+**	
No. of patients	41	49	45	45	180
No. of samples, serum pertuzumab^a^	NA	99	85	89	273
Age (years)	51 (37–74)	50 (28–74)	52 (22–68)	49 (27–70)	50 (22–74)
Race (a/b/h/w/m/i)^b^	11/0/0/29/1/0	10/1/0/37/0/1	8/0/0/37/0/0	8/1/1/33/1/1	37/2/1/136/2/2
Baseline weight (kg)	64.2 (40.5–102)	61 (45–99.1)	68 (35–104.9)	61.7 (44–90)	63.6 (35–104.9)
LBW (kg)	44.4 (32.2–52.7)	43.8 (36.7–55.5)	45.6 (29.3–55.8)	43.9 (33.5–56.1)	44.6 (29.3–56.1)
Albumin (g/dL)	4.2 (3.6–5)	4.4 (3.1–5)	4.4 (3.6–5)	4.4 (3.8–5.3)	4.4 (3.1–5.3)
SGPT (IU/L)	17 (3–49)	17 (6–72)	18 (5–56)	20 (11–43)	18 (3–72)
pCR (NA/No/Yes)	2/30/9	0/24/25	4/35/6	1/35/9	7/124/49

Continuous covariates were shown as median (range)

*LBW* lean body weight; *NA* not applicable; *pCR* pathological complete response; *No* number

^a^Included 15 predose serum pertuzumab samples (5 in Arm B, 4 in Arm C and 6 in Arm D). Predose samples were not used for the PK analysis

^b^Asian/Black/Hispanic/White/Mixed/Indian or Alaska native

The objectives of this analysis were to: (1) compare pertuzumab PK between the neoadjuvant population (early breast cancer [EBC]) in NeoSphere to a population of patients with tumor types including the first-line metastatic breast cancer (MBC) population, (2) to further explore the potential impact of trastuzumab and docetaxel on pertuzumab PK, and (3) perform an exposure–response (E–R) analysis to explore whether an E–R trend existed at the administered pertuzumab dose to further support selection of the clinical dosing regimen in the target patient population.

## Materials and methods

### Data included in the analysis

Pertuzumab serum concentrations were assessed in this study using optional biomarker sample repository (BSR; voluntary consented samples) blood samples collected on Days 14–21 (window of collection requested) post-dose on Cycles 2 and 4, based on the informed consent form (ICF). The trial was conducted in full accordance with the guidelines for Good Clinical Practice and the Declaration of Helsinki and met local institutional requirements and standards for clinical research. All patients provided written informed consent. Details of the study design of the NeoSphere trial have been described [6]. All NeoSphere patients who had pertuzumab serum concentration data available during Cycle 2 and/or Cycle 4 were included in the PK analysis, and all patients with available pertuzumab serum concentration data from Cycle 2 and/or Cycle 4 as well as pCR assessments from Cycle 4 were included in the exposure–response analysis. BSR blood samples were obtained from 180 patients: Arm A, *n* = 41; Arm B, *n* = 49; Arm C, *n* = 45; and Arm D, *n* = 45; patients in Arm A by design did not receive pertuzumab treatment. A validated enzyme-linked immunosorbent assay (ELISA) that allowed the quantification of pertuzumab in the presence of trastuzumab was used for the analysis of the samples in this study [12]. The minimum quantifiable serum concentration in human serum was 0.150 μg/mL for pertuzumab.

### Pharmacokinetic analysis

The pertuzumab serum concentration data collected in NeoSphere were analyzed using the published pertuzumab population PK model [14]. In this population PK model, pertuzumab PK was described by a two-compartment linear model with a clearance (CL), central volume of distribution (Vc), and terminal elimination half-life of 0.235 L/day, 3.11 L, and 18 days, respectively. Lean body weight (LBW) and baseline serum albumin concentration were identified as statistically significant covariates influencing pertuzumab PK.

To assess the agreement of the observed PK data in NeoSphere with the historical PK data based on the population PK model, a visual predictive check (VPC) and numerical predictive check (NPC) were performed. In the VPC, a total of 1000 trial replicates were simulated using the observed covariates (LBW and baseline albumin) and dose regimens for each patient, the model parameter estimates, and simulated patient-specific random effects. In the NPC, 1000 replicates were simulated for each patient using patient-specific covariates, dose regimens, and random inter-individual variability.

PK DDIs between pertuzumab and trastuzumab together with the docetaxel effect on pertuzumab PK were examined by comparing pertuzumab *C*_trough_ as well as individual PK parameters between different treatment groups. Pertuzumab individual PK parameters (i.e., empirical Bayesian estimates, EBEs) of the NeoSphere patients were generated using the population PK model. Analysis of variance (ANOVA) was used to compare PK parameters among different treatment groups, using a *p* value <0.01 as criteria of significance.

### Exposure–response analysis

The exposure–response relationship was evaluated between *C*_trough_ serum pertuzumab concentrations and pCR response. Observed and model-predicted serum *C*_trough_ at Cycle 2 were used as a measure of exposure as more patients had observed PK measurements in that cycle compared to Cycle 4. Given that samples for pertuzumab PK were collected between Days 14 and 21 post-dose, model-predicted Cycle 2 *C*_trough_ provided less variable results and therefore were used for the primary analysis.

The impact of pertuzumab exposure on pCR response was evaluated using a logit response model with a linear drug effect model according to:

$${\text{Log}}\left( {P/\left( {1 - P} \right)} \right) \, = \, E_{0} + {\text{Slope}} \times C_{\text{trough}}$$

where *E*_0_ is the pCR rate of the control group (Arm A) and Slope is the linear drug effect parameter. A slope that is significantly (*p* < 0.05) different from zero based on a log-likelihood ratio criterion would suggest a change of pCR response rate with exposure.

### Software

All data preparation, graphical presentations, and exposure–response analysis were performed using S-PLUS software, version 6.2 (TIBCO Software Inc., Palo Alto, CA). All PK analyses were implemented using NONMEM, version 7.1 (ICON Development Solutions, Hanover, MD).

## Results

In total, there were 180 patients with a BSR blood sample collected; 139 in the pertuzumab-containing arms (Arms B, C, and D) and 41 in the trastuzumab plus docetaxel arm (Arm A). Of the 180 patients, 173 had Cycle 4 pCR assessments available.

### Pertuzumab pharmacokinetic analysis

The pertuzumab Cycle 2 mean observed serum *C*_trough_ was 70 µg/mL with 98% (130 of 133) of patients in Arms B, C, and D achieving the PK target serum *C*_trough_ of > 20 µg/mL a PK target identified historically based on non-clinical data. The individual model-predicted mean *C*_trough_ at Cycle 2 was 60 µg/mL with 97% (130 of 134) of the patients achieving a predicted *C*_trough_ serum concentration of >20 µg/mL.

**Fig. 1:**
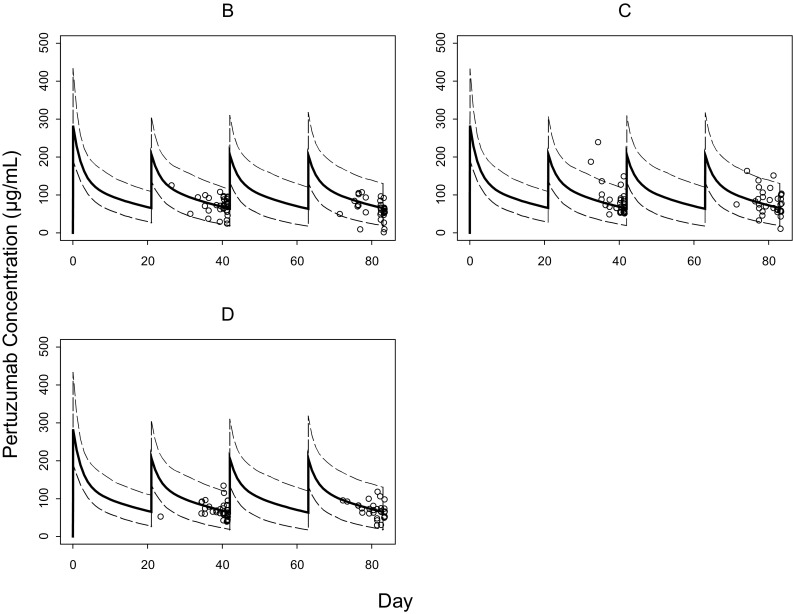
Observed versus simulated pertuzumab serum concentrations by treatment group. The *dashed lines* represent 97.5th and 2.5th percentiles based on simulations by the population PK model and the observed lean body weight and albumin distributions in NeoSphere. The *solid lines* are the population PK model predictions for a patient with the median values of lean body weight and albumin for each treatment group. The *open circles* represent *C*_trough_ serum concentrations observed for NeoSphere patients

The observed pertuzumab serum concentrations in NeoSphere matched the covariate-adjusted population PK model simulations as assessed by the VPC and NPC, demonstrating that the PK data in NeoSphere patients are comparable with PK data observed previously in patients with various metastatic solid tumors. The inter-individual variability of pertuzumab individual pharmacokinetic parameters by treatment groups is represented in Online Resource 1. NeoSphere patients had a slightly lower median LBW (44.6 vs. 49.2 kg) and a higher median serum albumin (4.4 vs. 3.9 g/dL) compared to the population used to build the pertuzumab population PK model and thus needs to be accounted for when comparing the PK. The VPC results are shown in Fig. 1, where the observed pertuzumab data (circles) in NeoSphere fall within the covariate-adjusted simulated pertuzumab concentrations for the historical population (thin lines) for each treatment arm. Overall, NPC suggests that the observed NeoSphere PK percentiles are in line the corresponding percentiles of the historical data as predicted by the model (95.7, 79.1, 39.9, 14.3, 5.0% for the 95, 75, 50, 25, 5th simulated percentile). The slight deviation in NPC can be attributable to the small sample size. The individual-predicted *C*_trough_ serum concentrations were well correlated with the observed *C*_trough_, supporting the use of model-predicted *C*_trough_ for DDI and ER assessment.

**Fig. 2:**
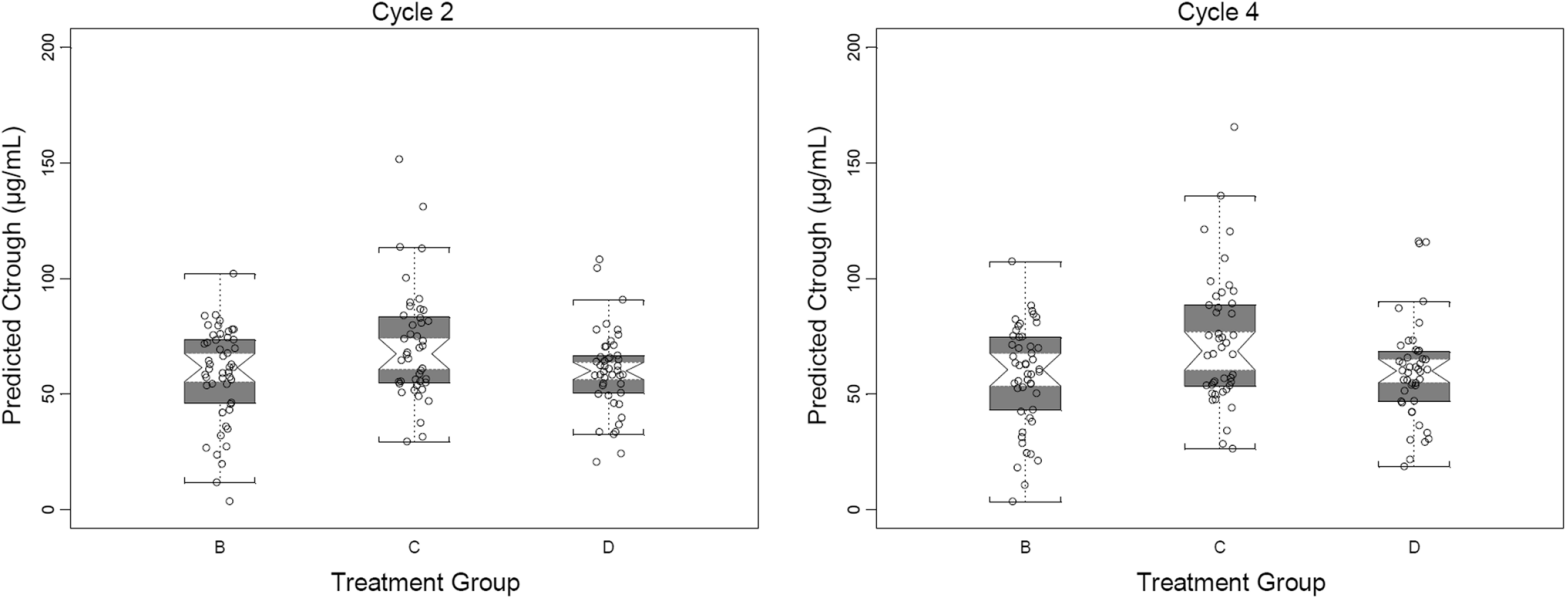
Predicted pertuzumab *C*_trough_ serum concentrations at Cycles 2 and 4. The *circles* represent predicted *C*_trough_ serum concentrations of individual patients, and the *squares* represent the mean value of the group. The *short lines* represent *C*_trough_ for a patient with the median values of lean body weight and albumin for each treatment group

PK DDIs between pertuzumab and trastuzumab together with the docetaxel effect on pertuzumab were assessed by comparing model-predicted *C*_trough_ serum concentrations at Cycles 2 and 4 as well as individual model-predicted pertuzumab PK parameters of NeoSphere patients in the different treatment groups. As shown in Fig. 2, the model-predicted *C*_trough_ concentrations appear similar across treatment groups, which were confirmed by ANOVA at either Cycle 2; *p* = 0.232 or Cycle 4; *p* = 0.039. The same analysis was performed using observed *C*_trough_ concentrations and yielded consistent results (Cycle 2, *p* = 0.458; Cycle 4, *p* = 0.033), Online Resource 2.

**Fig. 3:**
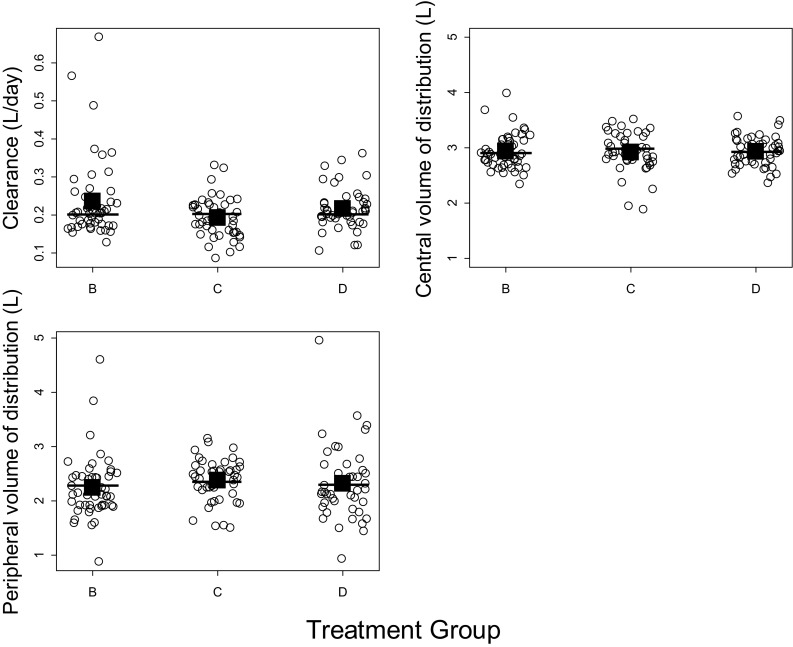
Pertuzumab individual pharmacokinetic parameters by treatment group. The *circles* represent pharmacokinetic parameters of individual patients, and the *squares* represent the mean value of the group. The *short lines* represent the parameters for a patient with the median values of lean body weight and albumin for each treatment group

The individual model-predicted pertuzumab PK parameters did not appear to differ between patients with or without trastuzumab as observed in Fig. 3. An ANOVA test confirmed that pertuzumab CL and Vc values were similar between patients with or without trastuzumab (*p* = 0.264 for CL and *p* = 0.956 for Vc, comparing Arms B and D) and patients with or without docetaxel (*p* = 0.016 for CL and *p* = 0.823 for Vc, comparing Arms B and C). Collectively the analyses showed no evidence of a DDI effect of trastuzumab on pertuzumab PK or of docetaxel in the presence of trastuzumab on pertuzumab PK.

## Exposure–response (ER) analysis

The ER population consisted of 173 patients that had both PK and pCR assessments available. Eighty-eight (88) patients from treatment groups A and B with week 4 pCR assessments were used in the ER analysis of pertuzumab. In each treatment group, the pCR rates of patients with PK results were similar to those of the overall treated patients for each arm of the study [6].

**Fig. 4:**
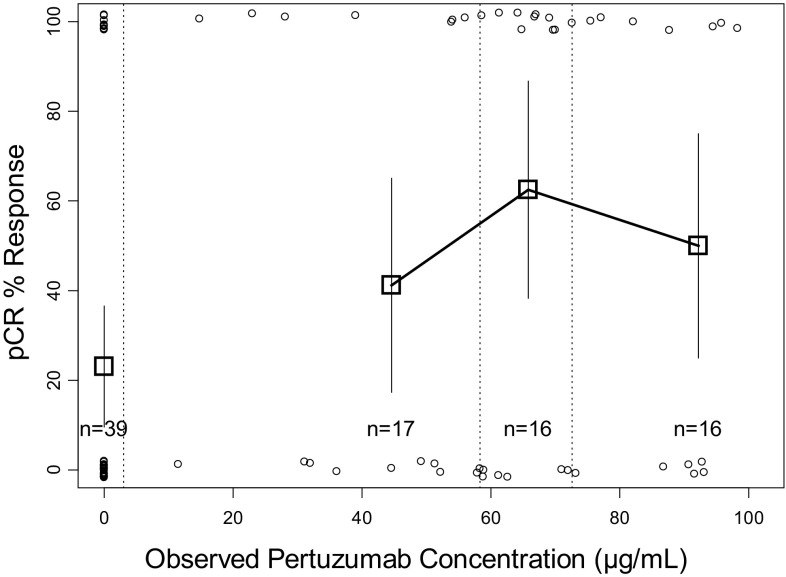
pCR response rate versus pertuzumab predicted *C*_trough_ serum concentrations (in combination with trastuzumab and docetaxel). *Square symbols* represent percent pCR (pathological complete response) of the patients grouped by pertuzumab serum concentration into third tiles (separated by *vertical dashed lines*). The group with zero pertuzumab serum concentration refers only to all patients in Arm A. *Error bars* represent 2 × standard error [2 × √(*p*×(1 − *p*)/*n*)]. The *open circles* represent the response status of individual patients (0% = non-responder, 100% = responder)

The pCR rate versus the model-predicted pertuzumab *C*_trough_ serum concentrations at Cycle 2 are illustrated in Fig. 4. The patients included in the plot comprised of two groups: Arm A treated with trastuzumab + docetaxel and Arm B treated with trastuzumab + docetaxel + pertuzumab combined together for the analysis. The model-predicted pertuzumab *C*_trough_ serum concentrations ranged from 3.4 to 103.2 μg/mL. Forty-six of 49 (94%) patients treated with pertuzumab (Arm B) had a predicted *C*_trough_ pertuzumab serum concentration of >20 μg/mL, the target efficacious exposure based on non-clinical efficacy models.

The pCR rate was higher in patients treated with pertuzumab plus trastuzumab and docetaxel compared with patient treated with trastuzumab and docetaxel (*p* < 0.05); however, there was no significant impact (*p* = 0.996) on the probability of pCR response with an increase in pertuzumab serum concentration (*C*_trough_) beyond 20 μg/mL (Fig. 4, panel A). An analysis using observed pertuzumab concentrations collected during Days 14–21 of Cycle 2 yielded very similar results (Online Resource 3).

## Discussion

The NeoSphere clinical trial was a four-arm study conducted to assess the activity of pertuzumab in neoadjuvant (EBC) setting with pCR as the primary efficacy end point (Table 1). In the NeoSphere study, a significantly higher proportion of women given neoadjuvant pertuzumab and trastuzumab plus docetaxel achieved pCR in the breast than did those given trastuzumab and docetaxel alone, with pCR rates of approximately 46% and 30%, respectively [6, 13]. The objectives of this analysis were to compare pertuzumab PK between the EBC population (neoadjuvant treatment) in NeoSphere and a population of patients with other tumor types including the first-line MBC population, to explore the potential impact of trastuzumab and docetaxel on pertuzumab PK, and to assess the relationship between exposure (*C*_trough_) and response (pCR) of pertuzumab (in combination with trastuzumab and docetaxel) in neoadjuvant treatment of EBC. pCR was selected as the outcome variable in our E–R analysis as it was the primary end point in the trial, later analyses showed a correlation between pCR and DFS/EFS, further supporting the utility of pCR as an end point. In the NeoSphere trial, the safety and tolerability of the triple regimen of pertuzumab, trastuzumab, and docetaxel were similar to those of trastuzumab plus docetaxel [15] and no unique safety signals were identified that could be attributed to pertuzumab exposure. Therefore, an ER analysis with respect to safety was not conducted.

As expected due to differences in demographics (100% females versus 62% females) and health status (EBC versus advanced solid tumors), the patients enrolled in the NeoSphere trial displayed LBW and albumin levels which were lower and higher, respectively, compared to the population PK model population, resulting in CL values that were slightly lower compared to the reference model. The PK model predictions of pertuzumab serum concentrations matched the observed serum concentrations after correcting for these baselines covariate differences. Pertuzumab PK in the EBC population in NeoSphere appear to be in agreement with the PK in patients with other tumor types, including the first-line MBC population, when adjusted for these characteristics.

The potential impact of trastuzumab or docetaxel on the pharmacokinetics of the pertuzumab was examined by comparing the individual model-predicted and observed *C*_trough_ and model-predicted PK parameters of pertuzumab among different treatment groups. The analyses showed that there was no evidence of impact of trastuzumab or of docetaxel in the presence of trastuzumab on the PK of pertuzumab. These results were not surprising since pertuzumab and trastuzumab are known to recognize different epitopes on the HER2 extracellular domain and do not compete for the same binding site [2, 16, 17]. Moreover, monoclonal antibodies (mAbs) and small molecules such as docetaxel are largely eliminated by distinct routes. Docetaxel is mainly metabolized by hepatic cytochrome P450 isoenzymes [18, 19], whereas mAbs are primarily eliminated through large-capacity, non-specific, Fc receptor-mediated IgG clearance mechanisms and through specific, target-mediated drug disposition pathways [20]. Additionally, these results are consistent with the results of a previous study that showed no DDI between pertuzumab and docetaxel and between pertuzumab and trastuzumab in the first-line MBC setting [12].

The pertuzumab clinical dosing regimen of a 840 mg fixed loading dose followed by 420 mg every three weeks was selected based on PK and safety data from studies where pertuzumab was administered as a single agent to patients with advanced refractory solid tumors, including ovarian cancer, metastatic breast cancer (low HER2 expressing), and hormone-refractory prostate cancer. Results from these clinical studies, from population pharmacokinetic analyses [21], and from dose–response studies in non-clinical xenograft models were used to determine the dose of pertuzumab used in late-stage clinical studies. In single ascending dose studies utilizing pertuzumab doses of 0.5–25 mg/kg [22, 23] and also in Phase II studies where patients were treated with either 420 mg q3w (following a loading dose of 840 mg) or 1050 mg q3w (with no initial loading dose) as a single agent or in combination with other chemotherapeutic agents, the maximum tolerated dose (MTD) was not reached and no clear dose–safety or dose–efficacy relationship was observed. In oncology, especially for chemotherapeutic agents, the MTD is often carried forward into Phase III studies. However, given that pertuzumab is a targeted monoclonal antibody and the MTD was not reached, the dose for Phase III studies was selected based on achievement of the clinical target pertuzumab concentrations (steady-state *C*_trough_ concentration of ≥20 µg/mL in 90% of patients) that resulted in maximal suppression of tumor growth in non-clinical xenograft dose–response studies [24]. In the NeoSphere study, the majority of patients (>90%) achieved the target serum concentrations. Consistent with the non-clinical xenograft studies, the ER analysis suggested that there was no association between pCR rate and pertuzumab concentrations within the observed concentration range of approximately 20–100 μg/mL, supporting no dose adjustments needed for patient with lower exposure. This analysis further supports the appropriateness of the fixed, non-weight-based pertuzumab dose of 840 mg followed by 420 mg q3w in the neoadjuvant treatment of early breast cancer patients.

## Electronic supplementary material

Below is the link to the electronic supplementary material.

Inter-Individual Variability of Pertuzumab Individual Pharmacokinetic Parameters by Treatment Group. The black circles represent the values of the individual patients. The lower and upper end of each box plot represents the 25th and 75th percentile, respectively, within each treatment group. Supplementary material 1 (DOCX 34 kb)

Observed Pertuzumab C_trough_ Serum Concentrations at Cycles 2 and 4. The circles represent observed C_trough_ concentration of individual patients, and the squares represent the mean value of the group. The short lines represent C_trough_ for a patient with the median values of lean body weight and albumin for each treatment group. Supplementary material 2 (DOCX 69 kb)

pCR Response Rate versus Pertuzumab Observed C_trough_ Serum Concentrations (in Combination with Trastuzumab and Docetaxel). Square symbols represent percent pCR of the patients grouped by pertuzumab serum concentration into third tiles (separated by vertical dashed lines). The group with zero pertuzumab serum concentration refers only to all patients in Arm A. Error bars represent 2 × standard error [2 × √(p*(1 - p)/n)]. The open circles represent the response status of individual patients (0% = non-responder, 100% = responder). Supplementary material 3 (DOCX 21 kb)

Observed versus model predicted (IPRED) pertuzumab concentrations. Cycles represent individual concentrations. Supplementary material 4 (DOCX 19 kb)
