# Supplementary material for: Pharmacokinetic and exposure–response analyses of pertuzumab in combination with trastuzumab and docetaxel during neoadjuvant treatment of HER2+ early breast cancer
Source: Cancer Chemother Pharmacol. 2017 Jan 10;79(2):353–61. doi: 10.1007/s00280-016-3218-0 (PMC5306091; doi:10.1007/s00280-016-3218-0)
Supplement: Supplementary file 1 — Inter-Individual Variability of Pertuzumab Individual Pharmacokinetic Parameters by Treatment Group. The black circles represent the values of the individual patients. The lower and upper end of each box plot represents the 25th and 75th percentile, respectively, within each treatment group. Supplementary material 1 (DOCX 34 kb) [file 280_2016_3218_MOESM1_ESM.docx]

Figure was created using Splus version 8.2
